# Genetic characteristics and clinical-specific survival prediction in elderly patients with gallbladder cancer: a genetic and population-based study

**DOI:** 10.3389/fendo.2023.1159235

**Published:** 2023-04-21

**Authors:** Hao Zhou, Junhong Chen, Hengwei Jin, Kai Liu

**Affiliations:** Department of Hepatobiliary and Pancreatic Surgery II, General Surgery Center, The First Hospital of Jilin University, Changchun, China

**Keywords:** energy metabolism, cell cycle, nomogram, elderly gallbladder cancer, OS, CSS

## Abstract

**Background:**

Biliary system cancers are most commonly gallbladder cancers (GBC). Elderly patients (≥ 65) were reported to suffer from an unfavorable prognosis. In this study, we analyzed the RNA-seq and clinical data of elderly GBC patients to derive the genetic characteristics and the survival-related nomograms.

**Methods:**

RNA-seq data from 14 GBC cases were collected from the Gene Expression Omnibus (GEO) database, grouped by age, and subjected to gene differential and enrichment analysis. In addition, a Weighted Gene Co-expression Network Analysis (WGCNA) was performed to determine the gene sets associated with age grouping further to characterize the gene profile of elderly GBC patients. The database of Surveillance, Epidemiology, and End Results (SEER) was searched for clinicopathological information regarding elderly GBC patients. Nomograms were constructed to predict the overall survival (OS) and cancer-specific survival (CSS) of elderly GBC patients. The predictive accuracy and capability of nomograms were evaluated through the concordance index (C-index), calibration curves, time-dependent operating characteristic curves (ROC), as well as area under the curve (AUC). Decision curve analysis (DCA) was performed to check out the clinical application value of nomograms.

**Results:**

Among the 14 patients with GBC, four were elderly, while the remaining ten were young. Analysis of gene differential and enrichment indicated that elderly GBC patients exhibited higher expression levels of cell cycle-related genes and lower expression levels of energy metabolism-related genes. Furthermore, the WGCNA analysis indicated that elderly GBC patients demonstrated a decrease in the expression of genes related to mitochondrial respiratory enzymes and an increase in the expression of cell cycle-related genes. 2131 elderly GBC patients were randomly allocated into the training cohort (70%) and validation cohort (30%). Our nomograms showed robust discriminative ability with a C-index of 0.717/0.747 for OS/CSS in the training cohort and 0.708/0.740 in the validation cohort. Additionally, calibration curves, AUCs, and DCA results suggested moderate predictive accuracy and superior clinical application value of our nomograms.

**Conclusion:**

Discrepancies in cell cycle signaling and metabolic disorders, especially energy metabolism, were obviously observed between elderly and young GBC patients. In addition to being predictively accurate, the nomograms of elderly GBC patients also contributed to managing and strategizing clinical care.

## 1 Introduction

Gallbladder cancer (GBC) is a kind of carcinoma mainly derived from gallbladder secretory cells; hence adenocarcinoma is the absolute dominant category. It is the predominant malignancy in the biliary duct system, making up more than 95% of cases (1). As reported by GLOBOCAN 2020, GBC ranks as the 25th most prevalent cancer and has a global mortality rate of 0.9% (2). However, the mortality of GBC (average 0.09%) is far lower than that of other highly malignant tumors, like lung cancer (18%) or female breast cancer (15%), the prognosis of GBC remains unsatisfying, which is possibly associated with non-specific manifestations, absence of early diagnosis and highly invasiveness of tumor itself (1, 3, 4). Nearly 1 out of 5 patients with GBC got timely diagnosis and treatment in the US (5). Because of characteristics like the peculiar anatomic site and blood supply of gallbladder, patients’ physical differences and heterogeneity of cancer cells, etc., GBC is not well responsive to traditional chemotherapy and radiotherapy; as a result, surgical resection remains the primary treatment approach for individuals diagnosed with GBC (6). Possibly due to GBC taking decades for full development, a majority of patients are old (≥ 65), and GBC is typically diagnosed at an average age of 72 in the US (5). The SEER database revealed that the incidence rates of GBC (per 100,000) were age-adjusted and varied by age group in 2015. The rates increased with age, from 0.2 for those aged 20-49 years, to 1.6 for those aged 50-64 years, to 4.3 for those aged 65-74 years, and to 8.1 for those aged over 75 years. The mortality rates (per 100,000) followed a similar pattern, rising from 0.1 for those aged 20-49 years, to 0.7 for those aged 50-64 years, to 2.1 for those aged 65-74 years, and to 4.9 for those aged over 75 years (7). This informed us that older people are a high-risk population for GBC and, in the meanwhile, for patients with GBC, the older they are, the poorer prognosis they may suffer. Therefore, it is crucial to exploit innovative biomarkers or robust models for predicting survival probability of elderly patients (≥ 65) with GBC to aid clinical management better.

Nomograms are digital graphical tools with the integration of several key variables, which are now commonly applied for event prediction, especially for prognosis prediction in cancers. Compared to the traditional TNM stage, it can include more tumor characteristics and has gained extensive usage in forecasting the outcomes of various cancer types (8–10). Several nomograms have been established for prognosis prediction, lymph node metastasis prediction, or distant metastasis prediction in GBC (11–16). Still, there are no nomograms that are exploited based on elderly patients with GBC. Owing to the specificity of the elderly patients, creating a new model for this group is necessary. The GEO database (https://www.ncbi.nlm.nih.gov/geo/) is a widely used gene sequencing database from which we retrieved 14 cases of GBC with age-specific characteristics. Therefore, this study investigated the genetic characteristics of elderly GBC patients based on the sequencing data from the GEO database. The SEER database (https://seer.cancer.gov/) is a reliable and thorough online resource for collecting cancer statistics from the US population. With the goal of assisting clinical decision-making and maximizing benefits, our aim was to pinpoint prognostic factors and construct a trustworthy nomogram for calculating the likelihood of survival in elderly GBC patients relying on the SEER database.

## 2 Materials and methods

### 2.1 RNA-seq data collection and analysis

The present study employed RNA-seq data from 14 GBC patients, sourced from two chips available in the GEO database, namely GSE62335 and GSE76633. In order to eliminate batch effects, all RNA-seq data were de-identified using the Combat method, and log2 normalization was performed, following the protocol of prior studies (17, 18). The young and elderly subgroups were defined by an age cutoff of 65 years, with 10 and 4 patients respectively. To identify differentially expressed genes between the two subgroups, limma was employed with the screening criteria of |log2FC| > 1 and *p*-value < 0.05 (19). Gene Set Enrichment Analysis (GSEA) was then performed using the Gene Ontology (GO), Kyoto Encyclopedia of Genes and Genomes (KEGG), and ReactomePA pathway gene sets. Additionally, Gene Set Variation Analysis (GSVA) was performed with the KEGG gene set as the reference gene set (20). WGCNA was applied to the differential genes between the two subgroups to further explore gene sets associated with aging (21). The important gene sets were annotated with gene function and Protein-Protein Interaction (PPI) analysis, and the top 10 hub genes in the PPI network were identified using the cytoHub method. To assess immune cell infiltration in the tumor microenvironment of the 14 GBC patients, immune cell prediction algorithms of the TIMER2.0 platform were employed (22). Finally, drug sensitivity and immunotherapy sensitivity analyses were conducted using the oncoPredict R package and TIDE analysis, respectively (23). The TIDE analysis for evaluating immunotherapy sensitivity is based on the TIDE website (http://tide.dfci.harvard.edu). The TIDE value obtained from the analysis can be used to assess the efficacy of immunotherapy. Generally, a higher TIDE value indicates lower sensitivity to immunotherapy.

### 2.2 Cohorts formation and data collection

The primary patient cohort was acquired from the SEER database (site code C23.9), including all patients diagnosed with GBC between 2010 and 2017. Inapplicable patients were screened out. Exclusion principles were detailed as follows (1): young patients (< 65) (2), without a pathological diagnosis (3), unknown tumor grade (4), unknown TNM stage (5), unknown tumor size (6), unknown surgery information and (7) survival period of under one month or indeterminate duration of survival.

Following exclusion, the training and validation cohorts were assigned at random in a 7/3 split. The SEER database provided clinicopathological information, which encompassed age, race, marital status, tumor size, gender, tumor grade, AJCC TNM stage, surgery information, radiotherapy, chemotherapy, overall survival (OS), and cancer-specific survival (CSS). The workflow is demonstrated in **Supplementary Figure**.

### 2.3 Nomograms construction and validation

The training cohort was subjected to both univariate and multivariate Cox regression analyses to identify independent prognostic variables. The resulting significant variables from the latter were then utilized to create nomograms for predicting CSS and OS, respectively. To assess the effectiveness of the nomograms, various methods were utilized. Calibration curves were used to display the accuracy of the predictions made by the nomograms. Meanwhile, time-dependent receiver operating characteristic (ROC) curves and area under the curve (AUC) were employed to evaluate how well the nomograms were able to distinguish between different groups over time. In order to ensure the validity of the results, the nomograms were then tested in a validation cohort, and the analyses were reperformed accordingly.

### 2.4 Clinical associations

Decision curve analysis (DCA) was performed to assess the suitability of the nomograms for practical clinical use in contrast to the AJCC TNM stage. Nomograms were utilized to calculate the optimal cut-off value for the risk score via the ROC curve for each patient. After calculating the risk scores, patients in the training and validation cohorts were classified into high-risk and low-risk categories. To evaluate the survival differences, we utilized K-M survival curves to analyze both CSS and OS between these groups in both cohorts. Additionally, we investigated the impact of various surgery conditions on survival differences for both high-risk and low-risk patients.

### 2.5 Statistical analysis

To compare between groups, either chi-square tests or non-parametric U tests were employed. Frequency distribution (%), obtained through the chi-square test, was used to describe the remaining variable types. The survival disparities between the groups were examined using the Log-rank test and K-M curves. The statistical analysis was conducted using R software (version 3.6.2). R packages utilized in this study included “rms,” “survival,” “survminer,” and “ggDCA.” All statistical significance in this study was determined using a *P*-value of ≤ 0.05.

## 3 Results

### 3.1 Gene differential analysis and gene enrichment analysis of GBC

**Figure 1:**
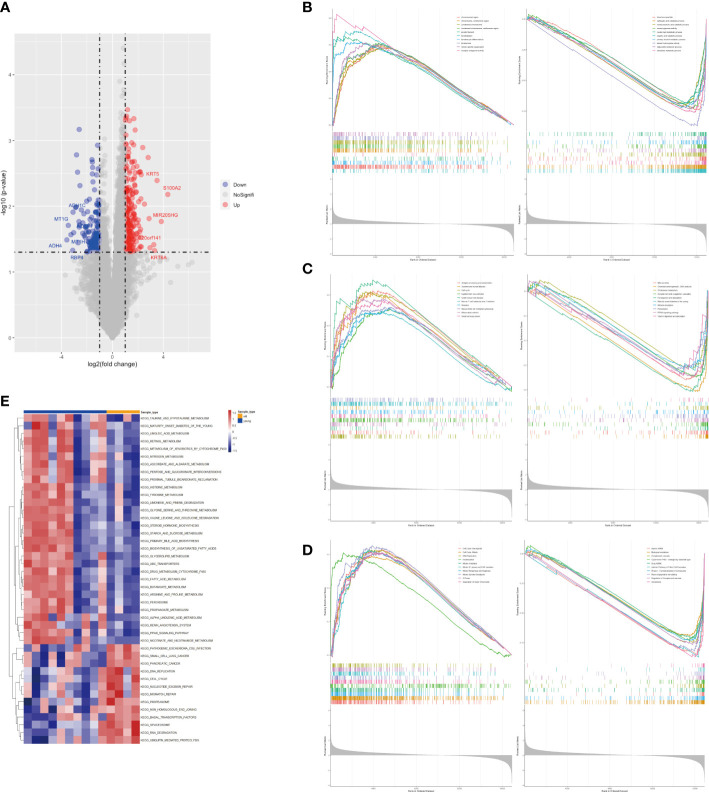
Gene differential analysis and gene enrichment analysis. **(A)** Volcano map of limma analysis. **(B)** GSEA enrichment analysis based on GO gene set. **(C)** GSEA enrichment analysis based on KEGG gene set. **(D)** GSEA enrichment analysis based on ReactomePA gene set. **(E)** GSVA pathway analysis based on KEGG gene set.

In this study, we enrolled a total of 14 patients with GBC and recorded their ages for further analysis. Through gene differential analysis, we identified 272 highly expressed genes and 150 lowly expressed genes in the elderly GBC group compared to the young GBC group (**Figure 1**). Further, using GSEA analysis based on the GO gene set, we observed an increased function of chromosomal and keratin-related genes and a decreased function of metabolism-related genes in elderly GBC patients (**Figure 1**). Similarly, GSEA analysis of differential genes based on the KEGG gene set showed an increased function of cell cycle-related genes and a decreased function of bile secretion-related genes in elderly GBC patients (**Figure 1**). Furthermore, the GSEA analysis based on the ReactomePA gene set showed an increased function of cell cycle-related genes and a decreased function of drug metabolism-related genes in elderly GBC patients (**Figure 1**). Finally, we performed GSVA analysis based on the KEGG gene set and found a decrease in metabolism-related pathways and an increase in cell cycle-related pathways in elderly GBC patients (**Figure 1**). By integrating the results of the above gene enrichment analyses, our study reveals a significant decrease in the expression of genes related to aerobic and lipid metabolism and an increase in the expression of genes related to cell cycle and mitosis in elderly GBC patients.

### 3.2 WGCNA analysis, drug sensitivity analysis, and immune microenvironment analysis of GBC

**Figure 2:**
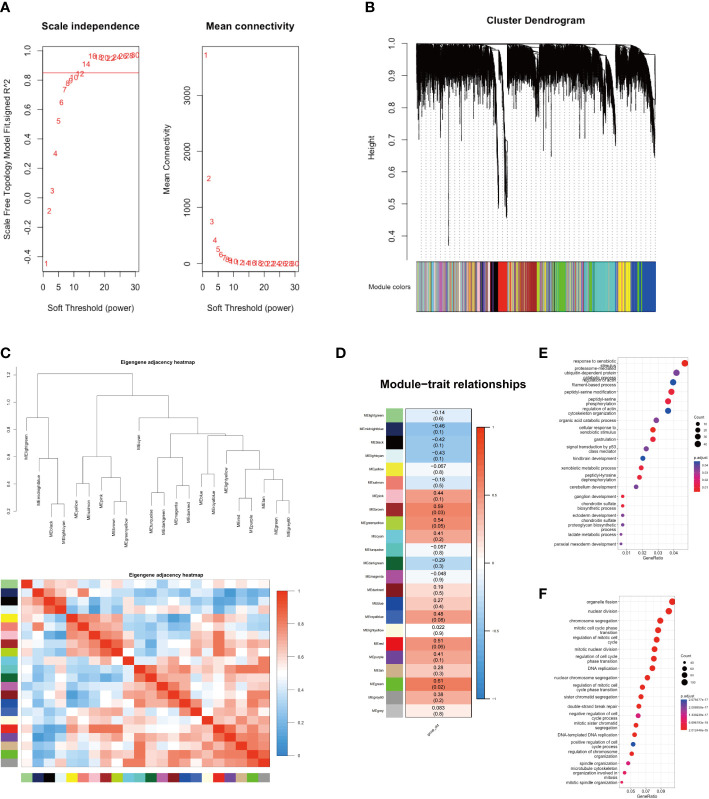
WGCNA analysis of two groups. **(A)** Scale independence and mean connectivity. **(B)** Cluster dendrogram of 22 modules. **(C)** Eigengene adjacency heatmap. **(D)** Module-trait relationships. **(E)** GO enrichment analysis of MEgreen gene module. **(F)** GO enrichment analysis of MEbrown gene module.

In this study, we conducted WGCNA analysis on sequencing data from two groups of GBC patients, employing a soft threshold of 14 (**Figure 2A**). We partitioned 13,991 genes into 22 gene set modules and subjected them to correlation analysis (**Figures 2B, C**). Our analysis revealed that the aging traits of GBC patients were significantly correlated with two gene modules, namely MEgreen (0.61, *p* = 0.02) and MEbrown (0.59, *p* = 0.03) (**Figure 2D**). Gene function annotation of the MEgreen gene module suggested that genes within this module were primarily associated with foreign body stimulation and aerobic metabolism (**Figure 2E**). On the other hand, functional annotation of the MEbrown gene module revealed that this module was mainly associated with the cell cycle and mitosis (**Figure 2F**). These findings corroborated our gene enrichment analysis results, demonstrating a low expression of energy metabolism-related genes and a high expression of cell cycle-related genes in elderly GBC patients.

**Figure 3:**
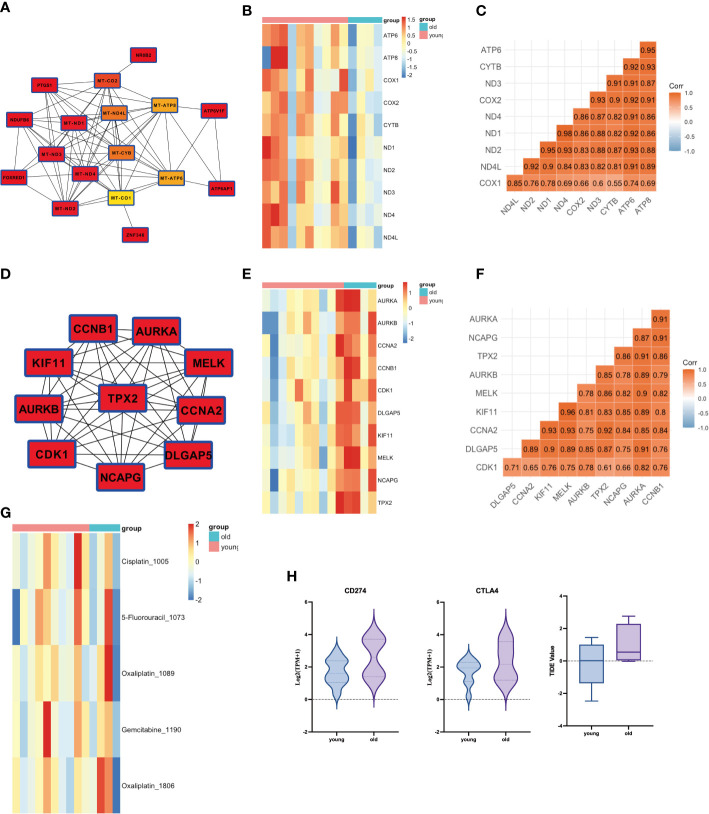
PPI analysis of WGCNA and drug sensitivity analysis. **(A)** Core network of the MEgreen gene module (MT: mitochondrial). **(B)** Gene expression of top 10 genes in MEgreen gene module. **(C)** Gene relation of top 10 genes in MEgreen gene module. **(D)** Core network of the MEbrown gene module. **(E)** Gene expression of top 10 genes in MEbrown gene module. **(F)** Gene relation of top 10 genes in MEbrown gene module. **(G)** Drug sensitivity analysis in common chemotherapy drugs. **(H)** Drug sensitivity analysis in immunotherapy.

We further performed a PPI analysis of the MEgreen and MEbrown gene modules and identified their top 10 hub genes (**Figures 3A**). The key hub genes, identified with the MT (mitochondrial) prefix, indicate that the majority of these genes originate from the mitochondrial genome (**Figure 3**). The top10 hub genes of MEgreen included ND1, ND2, ND3, ND4, ND4L, CYTB, COX1, COX2, ATP6, and ATP8, and the expression of these genes was reduced in elderly GBC patients (**Figure 3**). Moreover, they exhibited a high correlation (**Figure 3**). The top10 hub genes of MEbrown were mainly AURKA, AURKB, CCNA2, CCNB1, CDK1, DLGAP5, KIF11, MELK, NCAPG, and TPX2, and these genes were elevatedly expressed in elderly GBC patients (**Figure 3**) and had a high correlation (**Figure 3**). These results indicated that elderly GBC patients had a high expression of cell cycle-related genes and a low expression of mitochondrial respiratory enzyme-related genes, reflecting the genetic characteristics of elderly GBC that promote metastasis and deterioration of GBC cells.

**Figure 4:**
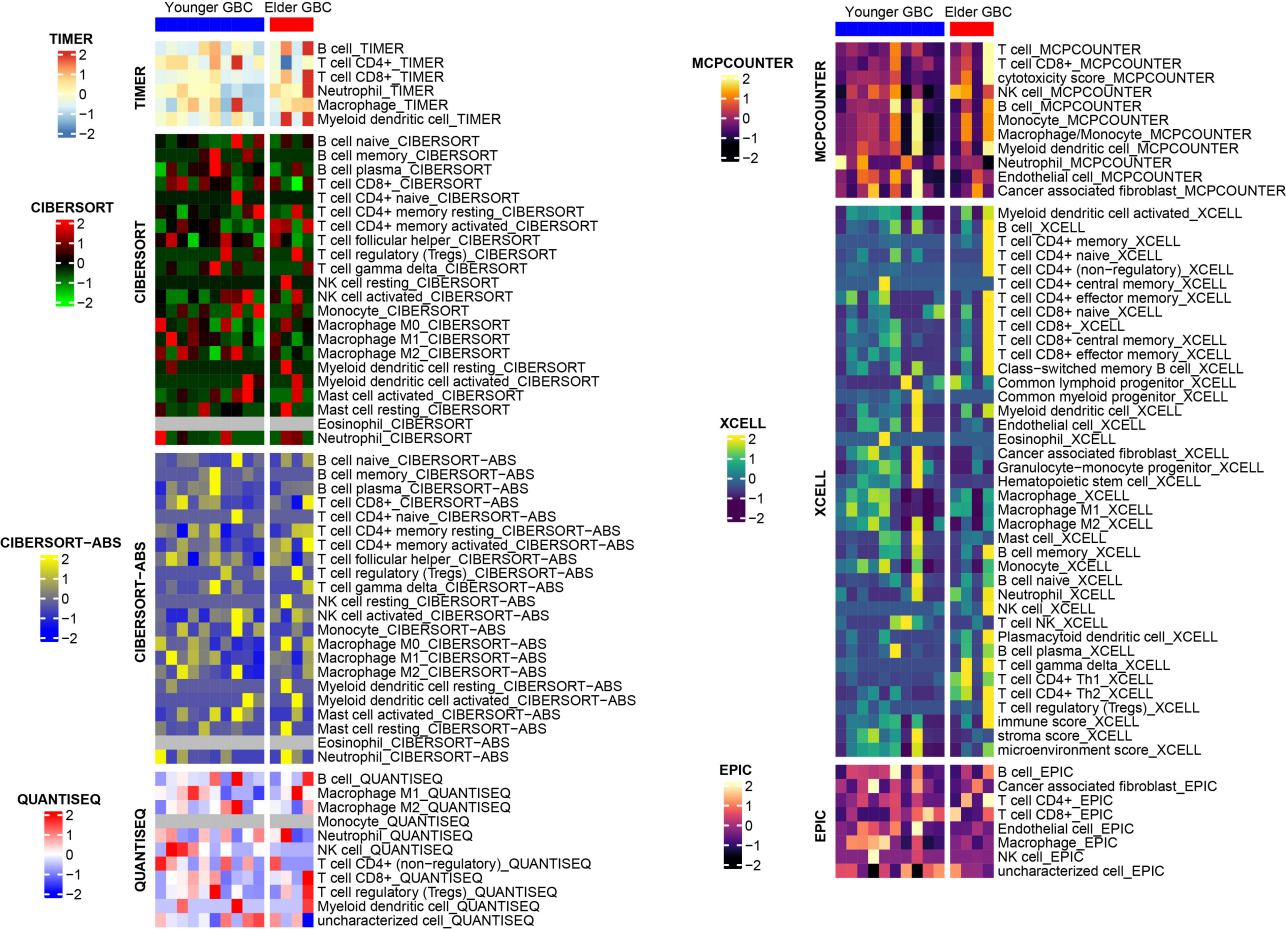
The discrepancies in immune cell infiltration between elderly and young GBC patients.

We also performed a drug sensitivity analysis for both groups, demonstrating that elderly GBC patients were less responsive to cisplatin and gemcitabine (**Figure 3**). Sensitivity analysis of immunotherapy revealed that elderly GBC patients displayed elevated expression of CD274 (PD-L1) and CTLA4, and demonstrated reduced responsiveness to immunotherapy, as indicated by a higher TIDE value (**Figure 3**). Finally, we employed a series of immunocyte prediction algorithms, which highlighted potential discrepancies in the tumor immune microenvironment between elderly and young GBC patients (**Figure 4**).

### 3.3 Clinicopathological characteristics of patients

8583 individuals with GBC between 2010 and 2017 were enrolled in the primary cohort. According to the exclusion criteria, 6452 inapplicable patients were screened out, including 2779 young patients (< 65), 866 patients without a pathological diagnosis, 1331 patients with unknown tumor grade, 436 patients with unknown TNM stage, 897 patients with unknown tumor size, 14 patients with unknown surgery information and 129 patients’ survival period of under one month or indeterminate duration of survival. As a result, 2131 elderly patients with GBC were finally identified and randomly allocated to the training cohort (1492, 70%) and validation cohort (639, 30%). The clinicopathological characteristics of enrolled patients in both cohorts were summarized in **Table 1**. Females accounted for 67.95% of all patients, while males accounted for 32.05%. 55.89% of patients have aged over 74, and 44.11% of patients are aged between 65 and 74. Patients who didn’t get married (51.99%) were approximately the same as patients who got married (48.01%). Most patients were white (77.48%). Tumor grades contained grade I (14.97%), grade II (44.91%), grade III (37.59%), and grade IV (2.53%). Patients were labeled as stage T1 (12.62%), stage T2 (47.82%), stage T3 (36.84%), and stage T4 (2.72%). Most patients were in the N0 stage (70.06%) and M0 stage (81.79%). Tumor size < 3 cm accounted for 49.37%, and tumor size ≥ 3 cm accounted for 50.63%. A majority of patients had local tumor excision/partial cholecystectomy surgery (82.40%), 11.78% of patients got radical cholecystectomy surgery, and only 5.82% of patients did not have surgery. A small part of patients (16.05%) got radiotherapy, and others (83.95%) did not. Also, the patients who had chemotherapy (34.58%) were less than those who did not (65.42%). There were no significant differences in these clinicopathological characteristics in both cohorts (*P* > 0.05).

**Table 1:** Clinicopathological characteristics of elderly patients with GBC.

	All N = 2131	Training Cohort N = 1492	Validation Cohort N = 639	*P*-value
**Sex**				0.591
Male	683 (32.05%)	484 (32.44%)	199 (31.14%)	
Female	1448 (67.95%)	1008 (67.56%)	440 (68.86%)	
**Age**				0.952
65-74	940 (44.11%)	657 (44.03%)	283 (44.29%)	
> 74	1191 (55.89%)	835 (55.97%)	356 (55.71%)	
**Marital status**				0.523
No	1108 (51.99%)	783 (52.48%)	325 (50.86%)	
Married	1023 (48.01%)	709 (47.52%)	314 (49.14%)	
**Race**				0.745
White	1651 (77.48%)	1160 (77.75%)	491 (76.84%)	
Black	211 (9.90%)	149 (9.99%)	62 (9.70%)	
Other	269 (12.62%)	183 (12.27%)	86 (13.46%)	
**Grade**				0.497
I	319 (14.97%)	223 (14.95%)	96 (15.02%)	
II	957 (44.91%)	685 (45.91%)	272 (42.57%)	
III	801 (37.59%)	548 (36.73%)	253 (39.59%)	
IV	54 (2.53%)	36 (2.41%)	18 (2.82%)	
**T stage**				0.817
T1	269 (12.62%)	185 (12.40%)	84 (13.15%)	
T2	1019 (47.82%)	709 (47.52%)	310 (48.51%)	
T3	785 (36.84%)	555 (37.20%)	230 (35.99%)	
T4	58 (2.72%)	43 (2.88%)	15 (2.35%)	
**N stage**				0.333
N0	1493 (70.06%)	1043 (69.91%)	450 (70.42%)	
N1	562 (26.37%)	401 (26.88%)	161 (25.20%)	
N2	76 (3.57%)	48 (3.22%)	28 (4.38%)	
**M stage**				0.402
M0	1743 (81.79%)	1213 (81.30%)	530 (82.94%)	
M1	388 (18.21%)	279 (18.70%)	109 (17.06%)	
**Tumor size**				0.773
< 3 cm	1052 (49.37%)	733 (49.13%)	319 (49.92%)	
≥ 3 cm	1079 (50.63%)	759 (50.87%)	320 (50.08%)	
**Surgery**				0.912
No	124 (5.82%)	88 (5.90%)	36 (5.63%)	
Local tumor excision/partial cholecystectomy	1756 (82.40%)	1226 (82.17%)	530 (82.94%)	
Radical cholecystectomy	251 (11.78%)	178 (11.93%)	73 (11.42%)	
**Radiotherapy**				0.694
No/Unknown	1789 (83.95%)	1249 (83.71%)	540 (84.51%)	
Yes	342 (16.05%)	243 (16.29%)	99 (15.49%)	
**Chemotherapy**				0.960
No/Unknown	1394 (65.42%)	977 (65.48%)	417 (65.26%)	
Yes	737 (34.58%)	515 (34.52%)	222 (34.74%)	

### 3.4 Independent prognostic predictors from Cox regression analysis

Next, in the training cohort, univariate Cox regression analyses were conducted to determine risk factors associated with OS and CSS, respectively. Detailed information was integrated in **Tables 2**, **3**. The results turned out to be that older age (> 74), higher tumor grade (grade II&III&IV), advanced TNM stage (T2&3&4, N1&2, and M1), and larger tumor size (≥ 3 cm) were significantly negatively correlated with OS. In contrast, married status and receiving surgery (local tumor excision/partial cholecystectomy surgery and radical cholecystectomy surgery) were positively correlated with OS. In terms of CSS, the risk factors mentioned above were still significantly associated with CSS, but age and marital status were. Receiving chemotherapy was mainly determined as a negative risk factor for CSS (HR = 1.267, 95% CI: 1.102-1.456). Results from subsequent multivariate Cox regression analyses further identified older age (> 74), married status, higher tumor grade (grade III&IV), advanced TNM stage (T2&3&4, N1, and M1), and receiving surgery (local tumor excision/partial cholecystectomy surgery and radical cholecystectomy surgery) as independent prognostic predictors for OS. Meanwhile, higher tumor grade (grade III&IV), advanced TNM stage (T2&3&4, N1, and M1), larger tumor size (≥ 3 cm), receiving surgery (local tumor excision/partial cholecystectomy surgery and radical cholecystectomy surgery), and receiving chemotherapy were determined as independent prognostic predictors for CSS.

**Table 2:** Univariate and multivariate Cox regression analysis of OS in training cohort.

	Hazard ratio	Univariate 95% CI	*P*-value	Hazard ratio	Multivariate 95% CI	*P*-value
Sex						
Male						
Female	0.972	0.858-1.101	0.651			
Age						
65-74						
> 74	1.339	1.188-1.510	< 0.001	1.535	1.356-1.737	< 0.001
Marital status						
No						
Married	0.869	0.772-0.978	0.019	0.851	0.754-0.960	0.009
Race						
White						
Black	0.992	0.815-1.207	0.933			
Other	0.851	0.706-1.026	0.090			
Grade						
I						
II	1.312	1.083-1.589	0.005	1.166	0.961-1.414	0.120
III	2.194	1.808-2.661	< 0.001	1.540	1.264-1.878	< 0.001
IV	3.059	2.097-4.463	< 0.001	2.053	1.397-3.017	< 0.001
T stage						
T1						
T2	1.670	1.334-2.090	< 0.001	1.556	1.239-1.955	< 0.001
T3	4.306	3.434-5.401	< 0.001	3.340	2.625-4.250	< 0.001
T4	8.008	5.533-11.592	< 0.001	5.373	3.643-7.923	< 0.001
N stage						
N0						
N1	1.599	1.404-1.820	< 0.001	1.215	1.058-1.395	0.006
N2	1.964	1.428-2.702	< 0.001	0.991	0.712-1.381	0.959
M stage						
M0						
M1	3.119	2.703-3.598	< 0.001	2.167	1.847-2.542	< 0.001
Tumor size						
< 3 cm						
≥ 3 cm	1.659	1.473-1.868	< 0.001	1.113	0.979-1.265	0.101
Surgery						
No						
Local tumor excision/partial cholecystectomy	0.249	0.199-0.313	< 0.001	0.658	0.513-0.845	0.001
Radical cholecystectomy	0.292	0.223-0.384	< 0.001	0.481	0.363-0.638	< 0.001
Radiotherapy						
No/Unknown						
Yes	0.927	0.794-1.084	0.344			
Chemotherapy						
No/Unknown						
Yes	1.069	0.946-1.209	0.283			

OS, overall survival; CI, confidential interval.

**Table 3:** Univariate and multivariate Cox regression analysis of CSS in training cohort.

	Hazard ratio	Univariate 95% CI	*P*-value	Hazard ratio	Multivariate 95% CI	*P*-value
Sex						
Male						
Female	0.909	0.788-1.049	0.193			
Age						
65-74						
> 74	1.135	0.988-1.302	0.073			
Marital status						
No						
Married	0.928	0.810-1.065	0.288			
Race						
White						
Black	0.954	0.756-1.204	0.692			
Other	0.834	0.670-1.038	0.104			
Grade						
I						
II	1.414	1.116-1.792	0.004	1.238	0.975-1.571	0.080
III	2.611	2.063-3.305	< 0.001	1.886	1.480-2.405	< 0.001
IV	4.244	2.825-6.376	< 0.001	2.676	1.765-4.058	< 0.001
T stage						
T1						
T2	1.708	1.287-2.265	< 0.001	1.653	1.241-2.203	0.001
T3	5.256	3.973-6.952	< 0.001	4.082	3.029-5.499	< 0.001
T4	10.345	6.809-15.718	< 0.001	7.635	4.913-11.866	< 0.001
N stage						
N0						
N1	1.787	1.542-2.071	< 0.001	1.397	1.187-1.643	< 0.001
N2	2.285	1.614-3.236	< 0.001	1.034	0.720-1.486	0.855
M stage						
M0						
M1	3.792	3.242-4.435	< 0.001	2.640	2.209-3.155	< 0.001
Tumor size						
< 3 cm						
≥ 3 cm	1.891	1.645-2.174	< 0.001	1.222	1.051-1.421	0.009
Surgery						
No						
Local tumor excision/partial cholecystectomy	0.214	0.168-0.273	< 0.001	0.671	0.513-0.878	0.004
Radical cholecystectomy	0.297	0.222-0.398	< 0.001	0.547	0.405-0.739	< 0.001
Radiotherapy						
No/Unknown						
Yes	1.004	0.841-1.198	0.965			
Chemotherapy						
No/Unknown						
Yes	1.267	1.102-1.456	0.001	0.570	0.485-0.669	< 0.001

CSS, cancer-specific survival; CI, confidential interval.

### 3.5 Construction of nomograms to predict OS and CSS at 1-, 3-, and 5-year

**Figure 5:**
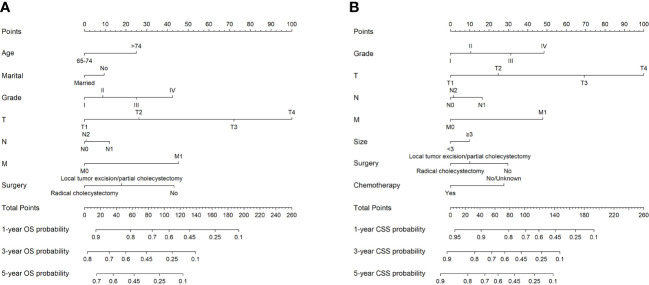
The nomograms to predict OS and CSS at 1-, 3-, and 5-year for elderly patients with GBC. **(A)** The nomogram to predict OS for elderly patients with GBC. **(B)** The nomogram to predict CSS for elderly patients with GBC.

Based on the results of Cox regression analysis, two distinct nomograms were created for predicting the OS and CSS at 1-, 3-, and 5-year, respectively (**Figure 5**). The nomograms revealed that certain demographic and clinical factors, such as age, marital status, tumor grade, surgery information, and TNM stage, played crucial roles in predicting OS. On the other hand, tumor grade, tumor size, surgery information, chemotherapy, and TNM stage were critical prognostic indicators for predicting CSS. In particular, T stage emerged as the most significant risk factor for both OS and CSS, as it had a considerable impact on the overall point score in the nomograms.

### 3.6 Validation of nomograms and performance evaluation

**Figure 6:**
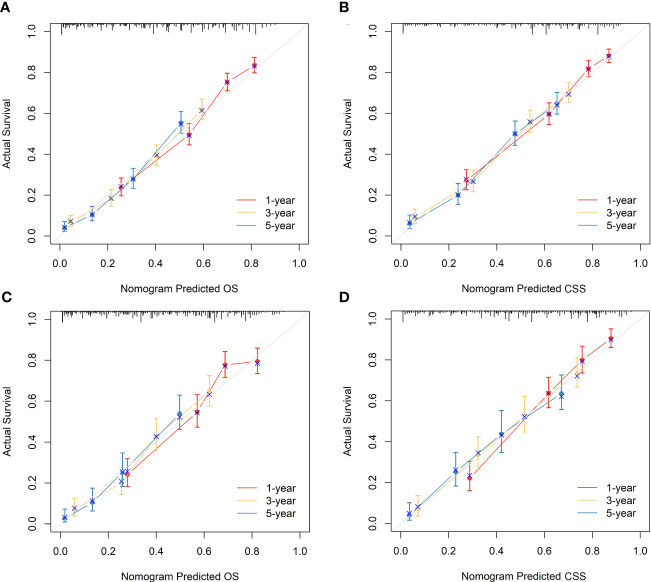
Calibration curves of the nomograms to predict OS and CSS at 1-, 3-, and 5-year for elderly patients with GBC. **(A)** Calibration curve of the nomogram to predict OS at 1-, 3-, and 5-year in the training cohort. **(B)** Calibration curve of the nomogram to predict CSS at 1-, 3-, and 5-year in the training cohort. **(C)** Calibration curve of the nomogram to predict OS at 1-, 3-, and 5-year in the validation cohort. **(D)** Calibration curve of the nomogram to predict CSS at 1-, 3-, and 5-year in the validation cohort. The horizontal axis of the nomogram represents the expected value, while the vertical axis represents the observed value.

**Figure 7:**
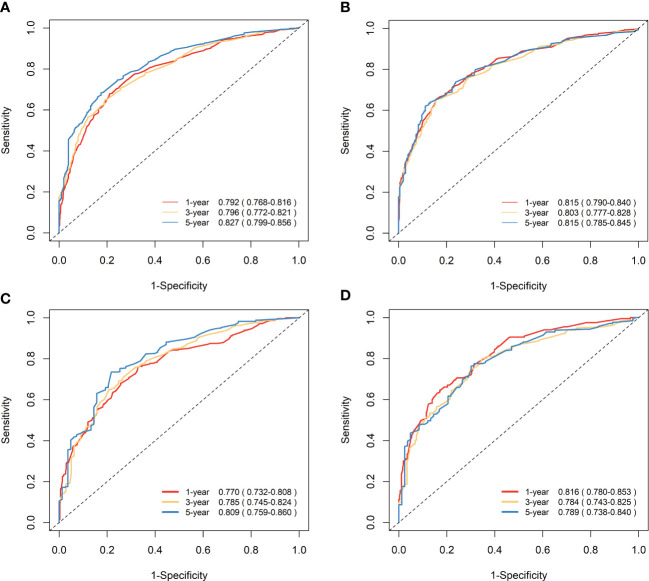
Time-dependent ROC curves to predict OS and CSS at 1-, 3-, and 5-year for elderly patients with GBC. **(A)** AUCs at 1-, 3-, and 5-year for OS prediction in the training cohort. **(B)** AUCs at 1-, 3-, and 5-year for CSS prediction in the training cohort. **(C)** AUCs at 1-, 3-, and 5-year for OS prediction in the validation cohort. **(D)** AUCs at 1-, 3-, and 5-year for CSS prediction in the validation cohort.

The nomograms were subjected to internal validation in the validation cohort, where the concordance index (C-index) was calculated. In the training cohort, the C-index for OS was 0.717 (95% CI: 0.701-0.732), and for CSS, it was 0.747 (95% CI: 0.730-0.764). The C-index for the validation cohort was also calculated for both OS and CSS, which were found to be 0.708 (95% CI: 0.682-0.733) and 0.740 (95% CI: 0.715-0.766), respectively. The C-index values for the validation cohort were found to be moderate, indicating a reasonable degree of accuracy for the nomograms. To assess the predictive performance of our nomograms, we utilized two evaluation methods: calibration curves and time-dependent ROC curves. The calibration curves, as depicted in **Figure 6**, demonstrated the accuracy of the predicted survival probabilities for both OS and CSS in both the training cohort and validation cohort. These curves exhibited a high degree of linearity, closely mirroring the actually observed survival probabilities. As such, our nomograms displayed robust predictive accuracy in both cohorts. Moreover, we also generated time-dependent ROC curves, as illustrated in **Figure 7**, to evaluate the discriminative ability of our nomograms. The AUCs for OS and CSS were calculated at 1-, 3-, and 5-year intervals for both the training and validation cohorts. The results revealed that our nomograms possessed excellent discrimination capabilities, with AUCs at 1-, 3-, and 5-year intervals ranging from 0.770 to 0.827 for OS and 0.784 to 0.816 for CSS across both cohorts. In summary, our nomograms demonstrated a high degree of accuracy in predicting survival probabilities, as evidenced by the calibration curves, and excellent discrimination capabilities, as indicated by the time-dependent ROC curves. These results support the robustness of our nomograms as a valuable tool for predicting survival outcomes.

### 3.7 Clinical application of nomograms

**Figure 8:**
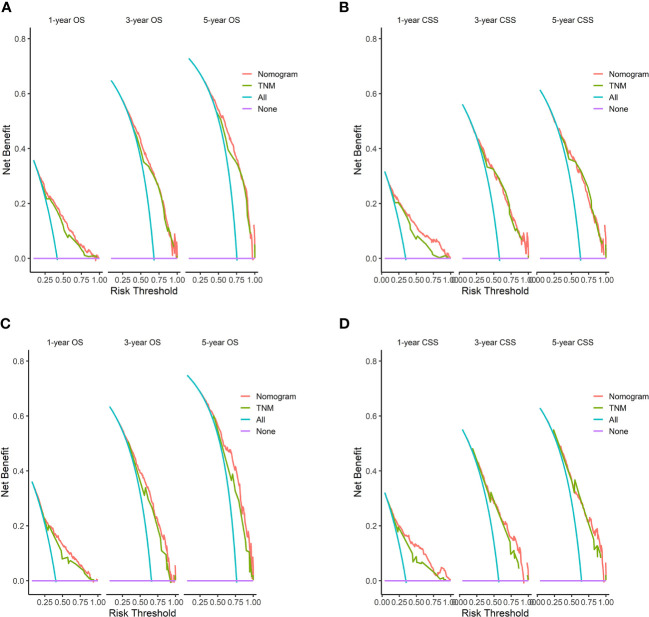
DCA of the nomograms to predict OS and CSS compared with TNM stage. **(A)** DCA of the nomogram to predict OS at 1-, 3-, and 5-year compared with TNM stage in the training cohort. **(B)** DCA of the nomogram to predict CSS at 1-, 3-, and 5-year compared with TNM stage in the training cohort. **(C)** DCA of the nomogram to predict OS at 1-, 3-, and 5-year compared with TNM stage in the validation cohort. **(D)** DCA of the nomogram to predict CSS at 1-, 3-, and 5-year compared with TNM stage in the validation cohort. When the threshold probability is between 20 and 100%, the net benefit of the model exceeds all deaths or none. DCA, decision curve analysis.

**Figure 9:**
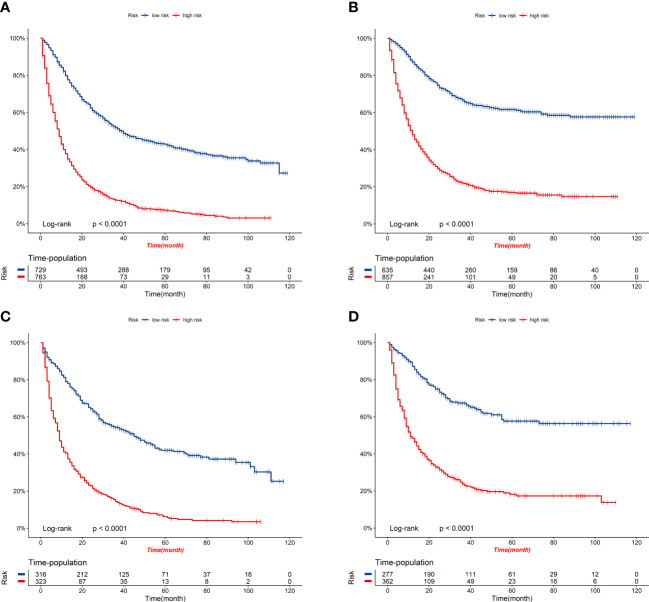
K-M survival curves of elderly patients with GBC in the high-risk and low-risk groups. **(A)** OS comparison of elderly patients with GBC based on risk score grouping in the training cohort. **(B)** CSS comparison of elderly patients with GBC based on risk score grouping in the training cohort. **(C)** OS comparison of elderly patients with GBC based on risk score grouping in the validation cohort. **(D)** CSS comparison of elderly patients with GBC based on risk score grouping in the validation cohort.

**Figure 10:**
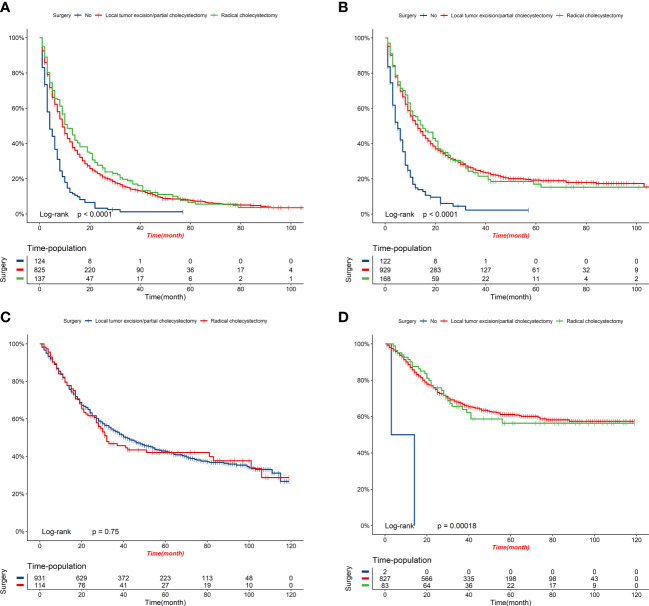
K-M survival curves of elderly patients with GBC under different surgery options. **(A)** OS comparison between patients under different surgery options in the high-risk group. **(B)** CSS comparison between patients under different surgery options in the high-risk group. **(C)** OS comparison between patients under different surgery options in the low-risk group. **(D)** CSS comparison between patients under different surgery options in the low-risk group.

**Figure 8** displays the outcomes of the DCA analysis, which showcases the superiority of our nomograms in terms of clinical benefits over the conventional TNM stage at 1-year in both the training and validation cohorts. However, the clinical benefits appeared to be out of advantage for our nomograms compared to the conventional TNM stage for 3-year and 5-year. This approved that our nomograms have better clinical application value to help clinicians assess early survival probability (1-year) both for OS and CSS compared to the conventional TNM stage. The nomograms were utilized to calculate the risk score and optimal cut-off value for each patient by means of the ROC curve. Patients were categorized into either a high-risk group, characterized by a total score greater than or equal to 96.00 for OS comparison and 88.91 for CSS comparison, or a low-risk group, with a total score less than the aforementioned cut-off values. The K-M survival curves demonstrated that patients who were classified as high-risk had a notably worse prognosis for both OS and CSS in both the training cohort and validation cohort, with all *P*-values being less than 0.0001 (**Figure 9**). For OS, the predicted survival probabilities at 1-, 3-, 5-year were 37.7%, 12.7%, and 7.1% for the high-risk group and 79.8%, 51.9%, and 42.8% for the low-risk group. For CSS, the predicted survival probabilities at 1-, 3-, 5-year were 48.1%, 21.9%, and 16.9% for the high-risk group and 87.3%, 66.8%, and 61.7% for the low-risk group. We found that the vast majority of patients underwent surgical treatment, both in the high- and low-risk groups. Moreover, survival probabilities (both OS and CSS) of patients who received surgery got significant improvements in contrast to that of patients who didn’t receive surgery in the high-risk group (*P* < 0.0001, **Figures 10A**). It appeared to be that receiving radical cholecystectomy surgery contributes to slight OS improvement in the early five years, compared to receiving local tumor excision/partial cholecystectomy surgery (**Figure 10**). However, it revealed no apparent difference in CSS improvement between the local tumor excision/partial cholecystectomy surgery subgroup and the radical cholecystectomy surgery subgroup (**Figure 10**). In the low-risk group, patients who received surgery got significant CSS improvement in contrast to that of patients who didn’t receive surgery. However, there were no significant survival improvements for both OS and CSS between the local tumor excision/partial cholecystectomy surgery subgroup and the radical cholecystectomy surgery subgroup (**Figures 10C**).

## 4 Discussion

In this study, we acquired GBC RNA-seq data from the GEO database and analyzed the genetic characteristics of elderly GBC patients. Our data set comprised 14 GBC patients, including four elderly and ten young patients. We performed genetic correlation analysis on age subgroups and discovered significant differences in the gene expression profiles of elderly and young GBC patients. Using WGCNA analysis, we identified a significant reduction in the expression of ND1, ND2, ND3, ND4, ND4L, CYTB, COX1, COX2, ATP6, and ATP8 genes, all of which are associated with mitochondrial respiratory enzyme functions in elderly GBC patients.

Additionally, pathway enrichment analysis results suggested that elderly GBC patients experience a significant decrease in aerobic metabolic processes, leading to reduced energy metabolism. Notably, few studies have investigated the metabolic aspects of GBC, making our findings particularly noteworthy. The reduced energy metabolic process observed in elderly GBC patients may hinder antitumor immune processes and drug metabolism, exacerbating the malignancy of aged GBC. Moreover, our study revealed an upregulation in the expression of cell cycle genes in elderly GBC patients, which could further contribute to their higher malignancy.

In addition, we successfully developed two nomograms to predict OS and CSS at 1-, 3-, and 5-year for elderly patients with GBC based on a large population from the SEER database. The predictive accuracy and capability of our nomograms were further verified in both the training cohort and validation cohort. Several independent prognostic predictors were identified and enrolled in our nomograms. Marital status, age, tumor grade, surgery information, T stage, N stage, and M stage were applied for OS prediction. Tumor grade, tumor size, chemotherapy, surgery information, T stage, N stage, and M stage were applied for CSS prediction.

Good experience and emotional support from marriage may positively help patients to struggle with cancer. Marital status has been determined as a protective risk factor of OS for patients with GBC (24). Accordingly, our results also identified married status as a protective prognostic predictor of OS for elderly patients with GBC, but the marital status was inapplicable for CSS prediction. Age appears to be a common risk factor for prognosis in many cancer types (25–30), as well as in GBC (12). Generally, the older patients are, the poorer prognosis they may suffer. We came to the same conclusion that elderly patients aged over 74 (> 74) have a poorer prognosis (both OS and CSS) in contrast to elderly patients aged no more than 74 (≥ 65, ≤ 74). Tumor grade and TNM stage are essential evidence for clinicians to evaluate the clinical outcomes of patients. Higher tumor grade and more advanced TNM stage underline enhanced malignant potentials of cancer cells, naturally inferring worse clinical outcomes. In accordance with previous studies (12, 31), we verified that patients with higher tumor grades and more advanced TNM stage were calculated with higher risk scores and worse prognoses (both OS and CSS). Notably, results from DCA revealed the superior advantage of our nomograms to predict OS and CSS at 1-year compared to the traditional TNM stage for elderly patients with GBC. There is no common standard for tumor size grouping in GBC. In previous studies, the cut-off points include 2 cm and 5 cm (32), 1.4 cm and 6.3 cm (33), 1.9 cm and 4.8 cm (24), 4.5 cm (34), and 5 cm (35), etc. In this present study, we selected 3 cm as the cut-off point. Although the standards vary, the results all pointed out that tumor size is associated with the prognosis of patients with GBC (24, 32–35). Particularly, Zhang et al. (33) and Yan et al. (35) reported that larger tumor size is negatively associated with CSS of patients with GBC, which was consistent with our finding.

The clinical treatment of GBC is a comprehensive strategy, with the chief component being surgical resection (6, 36). Currently, a combination of PD-1/PD-L1-based immunotherapy and traditional cytotoxic drugs is rising to be an option for first-line treatments (37). Radiotherapy is set as a postoperative treatment for patients with GBC, especially for those with lymph node involvement and positive resection margins (38). In this present study, we determined that surgical section does improve survival probabilities (both OS and CSS) of elderly patients with GBC in contrast to patients without surgical resection in the high-risk group. Further, radical cholecystectomy surgical resection may contribute to slight OS improvement in the early five years compared with local tumor excision/partial cholecystectomy surgical resection. In the low-risk group, no significant survival improvements were observed for both OS and CSS between the local tumor excision/partial cholecystectomy surgery subgroup and the radical cholecystectomy surgery subgroup. In other words, radical cholecystectomy surgical resection may not achieve more satisfying clinical benefits as we expected in contrast to local tumor excision/partial cholecystectomy surgical resection for patients in the low-risk group. This finding may provide evidence for the choice of operation types, and it is possible to provide more rational treatment management for patients based on their risk stratification. However, to the best of our knowledge, our nomograms were the first to analyze the associations between survival benefits and surgery options based on risk score grouping in elderly patients with GBC. Receiving chemotherapy was determined to be a protective prognostic predictor for CSS prediction of elderly patients with GBC (HR = 0.57, 95% CI = 0.485-0.669), which was consistent with reported results (39, 40).

Radiotherapy can serve as a valuable supplementary therapy for particular groups of patients, particularly those at a higher risk of recurring cancer, such as individuals who have undergone an R1 resection or those who have tested positive for lymph nodes. A study conducted previously demonstrated that the implementation of adjuvant radiotherapy resulted in an increased survival rate among patients who had been diagnosed with gallbladder cancer and were also affected by regional lymph node metastasis (41). However, in our study, radiotherapy was not an influential factor in the prognosis of elderly patients with GBC. It could be because elderly patients often cannot tolerate having radiotherapy or cannot obtain more benefits because of the combination of multiple underlying diseases.

Despite the robust predictive accuracy and capability of our nomograms, there were still several limitations of this present study. Above all, relevant treatment information, blood test data, and essential clinical characteristics of patients with GBC were not provided in the SEER database, such as chemotherapy regimens, radiation dose, blood routine tests, liver function, tumor markers, smoke, alcohol consumption, etc. Recruitment of these factors may help to optimize the predictive accuracy of nomograms. Second, multi-omics data are recommended to improve nomograms to emphasize precision medicine. Besides, our data are all from the U.S. population, and their applicability to populations in other countries remains to be verified, and additional multicenter prospective studies are needed to validate our findings.

## 5 Conclusions

Discrepancies in cell cycle signaling and metabolic disorders, especially energy metabolism, were obviously observed between elderly and young GBC patients. In addition to being predictively accurate, the nomograms of elderly GBC patients also contributed to managing and strategizing clinical care.

## Data availability statement

The original contributions presented in the study are included in the article/**Supplementary Material**. Further inquiries can be directed to the corresponding author.

## Ethics statement

Ethical review and approval was not required for the study on human participants in accordance with the local legislation and institutional requirements. Written informed consent for participation was not required for this study in accordance with the national legislation and the institutional requirements.

## Author contributions

All authors contributed to the article and approved the submitted version. In this study, all authors contributed significantly to the design, data collection, interpretation, and manuscript preparation and revision.

## Supplementary material

Supplementary Figure Workflow of the present study.
