# Supplementary figures and images for: Genetic characteristics and clinical-specific survival prediction in elderly patients with gallbladder cancer: a genetic and population-based study
Source: Front Endocrinol (Lausanne). 2023 Apr 21;14:1159235. doi: 10.3389/fendo.2023.1159235 (PMC10160488; doi:10.3389/fendo.2023.1159235)

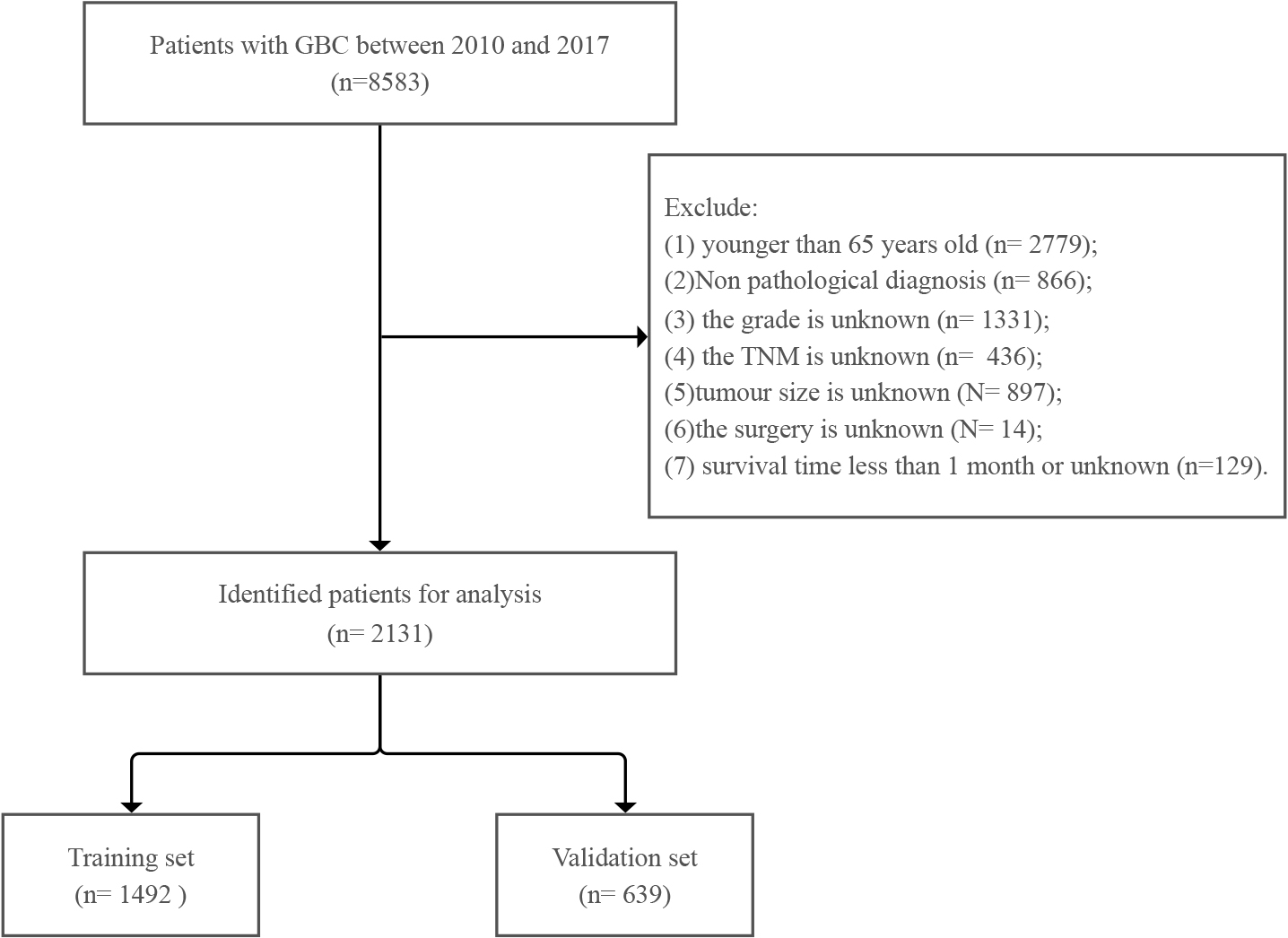

Supplement: Supplementary Figure — Workflow of the present study. [file Image_1.jpeg]
