# Supplementary material for: Genetic characteristics and clinical-specific survival prediction in elderly patients with gallbladder cancer: a genetic and population-based study
Source: Front Endocrinol (Lausanne). 2023 Apr 21;14:1159235. doi: 10.3389/fendo.2023.1159235 (PMC10160488; doi:10.3389/fendo.2023.1159235)
Supplement: Supplementary file 2 [file DataSheet_1.docx]

The race of patients was classified as white, black, and other types (Asian/Pacific Islander, American Indian/AK Native). The histological classification of tumors involved high differentiation (grade I), moderate differentiation (grade II), low differentiation (grade III), and undifferentiated (grade IV). Based on the SEER surgical code, we classified the surgical methods into non-surgical surgery (surgical code 0), local tumor resection/partial cholecystectomy (surgical code 10-50), and radical cholecystectomy (surgical code 60).

OS was defined as the duration from diagnosis to death or the final follow-up.

CSS was defined as the period between diagnosis and death from gallbladder cancer or the final follow-up.
